# Availability and access to Livelihood capital assets for development of sustainable Livelihood strategies of fishermen: A case study of Manchar Lake Pakistan

**DOI:** 10.1016/j.heliyon.2023.e22549

**Published:** 2023-11-25

**Authors:** Zhao Xu, Maria Qayum, Jamil Afzal, Muhammad Aslam

**Affiliations:** a College of Economics & Management Science, China Three Gorges University, Yichang, 443002, China; b College of Hydraulic & Environmental Engineering, China Three Gorges University, Yichang, 443002, China; c Department of Statistics, Faculty of Science, King Abdulaziz University, Jeddah, Saudi Arabia

**Keywords:** *Small scale fishermen*, *Livelihood assets*, *Household income*, *Sustainable livelihood*, *Poverty reduction*, *Manchar lake basin*

## Abstract

In developing nations with an abundance of natural water resources, fishing is a major source of income. When distributing resources between fisheries and other sectors, and preserving competitiveness in the fisheries industry, access to livelihood assets continues to be a key factor. However, vulnerability of livelihood assets and other economic and social variables have been severely affecting fishermen's livelihood. This article examines livelihood income vulnerability in the context of capital assets and the effects on local fishermen's livelihood and well-being. A survey sample size of 532 rural households based on a descriptive research design was used. Financial capital, human capital, social capital, and natural capital all made an enormous contribution to the earnings of fishermen and household heads in the Manchar Lake basin. The use of multiple regression analysis enabled new insights into how the livelihood assets characteristics affected their household income choices. The main objective of this investigation is to see whether access of livelihood capital assets affect local fishermen's livelihood income in the Manchar Lake basin. In the coastal belt of the Manchar Lake basin, resource acquisition plays a critical role in eradicating poverty. For the fishermen in the Manchar Lake basin, the current regime could further prioritize investing in fish trading infrastructure and improving essential facilities like social networks, education, skillsets, improved technology, and credit facilities in order to raise the livelihood income of fishermen and either reduce or alleviate poverty. These findings provide insight into fishermen's entry and exit for those who are planning livelihood shifts, and offer recommendations on how to overcome the constraints faced by the fisheries industry. Furthermore, insight into livelihood sustainability in order to improve the quality of life and income of fishermen domestically and nationally is also suggested.

## 1 Introduction

The sustainable livelihood strategy is an integrating component that enables initiatives to contribute to the simultaneous economy, conservation and sustainable management, and reduction of poverty. The concept is founded on the idea that to obtain a sustainable quality of life, individuals need a diverse combination of livelihood capital assets [1], for example, physical, financial, human, social, and natural assets. The Department for International Development of the United Kingdom established a sustainable livelihood framework, which was used in this study (DFID, 2000). The livelihood's assets and capabilities are the cornerstones of this strategy. The assets are similar to inputs that are used in several poverty-reduction schemes. Poverty among fishermen may be divided into two categories: income and non-income. People's competence and susceptibility make up the non-income aspect [2]. Livelihood resilience is a metric that assesses vulnerability aside from physical, natural, socioeconomic, and other factors. Capability refers to people's ability to employ livelihood assets. Capability focuses on the disadvantage of people's potential to achieve economic objectives. Abilities have predominantly been measured in terms of human wellbeing, such as average lifespan and health status. Poverty eradication, increased economic earnings, and unindustrialized production have always been the most important consequences for small-scale fishermen's livelihoods [3]. Physical, natural, human, social, and financial capitals were considered livelihood capital assets of fishermen in this study. Basic transportation and fishing instruments make up physical capital. Small-scale fishermen's physical assets include transportation, information flow, storage facilities, fishing boats and gear. Natural capital is the circulation of environmental asset endowments such as farmland, fishing grounds, ecology, and environmental impacts. These environmental assets provide the foundation for fishing operations and are critical to small-scale fishermen's socioeconomic outcomes. Capabilities, education, expertise, and excellent health are all examples of human capital, and human capital is critical to enhancing fishermen's capacity to explore various economic options. Small-scale fishermen's social capital comprises of their social networking, trustworthiness, relationships, affiliation, and management [4]. These social standards serve as the foundation for the coordination of each fishing society. Collaboration is a source of support for families of fishermen when it leads to achieving livelihood goals. Financial capital refers to accessible economic means such as deposits, loans, investments, insurance, and hereditary property [5]. By providing disposable income, adequate cash empowers fishermen with a variety of livelihood possibilities. Fishermen's ability to obtain diverse capital assets is critical to their survival. However, a particular asset type cannot sustain the livelihood goals of fishermen (see Fig. 3) (see. Fig. 2).

H = Human Capital, S = Social Capital, P = Physical Capital, F = Financial Capital, N = Natural Capital.

Y = Positive Influence of policy transformation.

Z = Negative Influence of policy transformation.

By providing disposable income, adequate cash empowers fishermen with a variety of livelihood possibilities. Fishermen's ability to obtain diverse capital assets is critical to their survival. However, a particular asset type cannot sustain the livelihood goals of fishermen [6]. Robert Chambers and Gordon Conway developed a theoretical reinforcement conceptual framework of a sustainable rural livelihood in 1992 which is generally practiced at the domestic level: a livelihood consists of expertise, capital (neighborhood markets, infrastructure, assertions, and availability), and improvement in the standard of living. The sustainability of livelihood should be realized if it can be figured out how to deal with and compensate against disruptions, retain or expand its functionalities and capital assets, and deliver a future sustainable livelihood. Furthermore, it provides financial yields through other livelihoods on a micro - and macro - level development in the near and distant future [7]. The spectrum of capital assets from which individuals build one ‘s lifestyle mostly encompasses household goods and possessions, as well as intangible belongings such as entitlements and accessibility. Therefore, it is perhaps one of the most complicated factors concerning a person's livelihood [8]. Natural, social, human, physical, and financial assets of livelihoods seem to be the five diverse kinds of capital assets that individuals keep in order to create an improved household environment. Acquisition of livelihood capital assets has become unswervingly linked in achieving a long-term sustainable livelihood. According to this observation, the policymakers have focused and highlighted their actions for this imperative goal [9].

The Tamron Japan Special Financing, which is funded by the Japanese government, is a comprehensive economic initiative to enhance the strategic planning of maritime conservation and sustainability in an attempt to optimize their status and reduce risks to inherent communities and ecosystems (Chen et al.). The initiative will encompass the maritime neighborhoods of Badin and Thatta as well as Karachi's coastal regions. The resourceful coastal belt surrounding Sindh territory's 350-km coastline contributes over 70 % of Pakistan's gross aquatic accumulation. The blue economy as well as coastal horticulture is the mainstay of the near-shore and deltaic households of Sindh, which have a population of roughly 2.3 million, of which a significant number are impoverished [10]. Unfortunately, a serious climate change deficit has harmed the neighborhood's indigenous flora, which is under continual threat due to sea level rise [11]. Relatively low mangrove covers and reproductive habitats for numerous economically valuable fish and invertebrates are some examples.

To increase family income levels in Pakistan, the authorities have initiated different steps, such as government planning and economic transformation planning, among others. In Pakistan, the Food and Agriculture Organization of the United States (FAO) performs a wide range of development and emergency aid initiatives. Projects are financed by the organization's own money, from bilateral and international assistance organizations, and from administrations, particularly the Pakistani government [12]. According to statistical information, the Asian Development Bank (ADB) has sanctioned technical assistance (TA) funding of US $650,000 to provide support to the Government of Pakistan in developing a plan to improve livelihood prospects and stimulate sustainable natural resource management in the coastal and inland populations of Sindh Province.

The main focus of this study is to assess a group of fishermen's livelihoods located in Pakistan's Indus River region's regarding income vulnerability and entitlement to assets. Manchar Lake was randomly selected to achieve this investigation goal, and a final sample size of 532 respondents was selected from the marginalized population inside the remote village of Manchar Lake and its surrounding area. Manchar Lake, often known as Manchar, is Pakistan's major ecological lake, and is among the world's largest. It is situated in Sindh, Westley, on the Indus River, inside the Dadu and Jamshoro localities, 18 km beyond Sehwan Sharif district [13]. Mega hydropower projects exert different effects on the surrounding and catchment areas. A comprehensive research instrument has been designed to predicate the Sustainability Livelihood Analysis (SLA) in order to gather enough data on rural livelihoods [14], which also include human, financial, social, natural, and physical capital assets [15]. In comparison to all the livelihood security vulnerabilities in the community in the Manchar Lake Basin, this research paper's findings indicate that human assets have been the most important asset among all other livelihood capital assets, and this provides a significant contribution to their livelihood [16]. The legislators who are intimately associated with the improvement of agrarian or remote settings would benefit from this investigation [17]. This research has provided a variety of solutions for improving the long-term viability of vulnerable groups' livelihood income.

This study aims firstly to close a significant research gap by identifying the factors driving the fisheries sector of Pakistan and evaluating the degree of fishermen's livelihood asset entitlement across livelihood income vulnerable groups with fishermen in Pakistan's Indus River region. Secondly, this study further investigates the impact of fishing activities on households' wellbeing in terms of total household income, food security status, and ability to save for emergencies. Thirdly, this study also aims to identify the beginner fishermen—who are they, and why did they choose fishing for their livelihood income? To the best of our knowledge, this is the first empirical work to examine the impact of access to livelihood assets on fishermen's households' income decisions in rural areas of Pakistan. To achieve the goals above, this study is organized as follows: Section 2 provides a comprehensive literature review, followed by an introduction to fishermen's livelihood options in Pakistan. Section 3 provides a conceptual framework and hypothesis development, followed by an introduction to our methods and data. Section 4 provides estimated results and analyses, followed by a balance test and sensitive analyses. The last section provides the conclusion and recommendations.

## 2 Review of the literature

### 2.1 Manchar Lake fisheries undertaking

The fishing industry significantly contributes to the financial welfare of people living in wetlands by providing employment, food and nutrition surveillance [18]. Local towns and villages in coastal areas rely on aquaculture for both survival and income, including nutrition, housing, and higher living standards [19]. Approximately 90 % of fishermen work in small-scale aquaculture. In terms of livelihoods, earnings, food and nutrition security, the segment is the largest source of revenue [20]. Fisheries supplies contribute up to 70 % of animal protein, and the industry employs more than 70 % of the people in the coastal zone in Sindh Province, namely around Manchar Lake [21]. Manchar Lake has a diversified fish habitat, with carps, catfish, snakeheads (murals), spinyeels, and tilapia being the most common. There are 32 species reported, 13 of which are considered commercially valuable species. According to the latest statistics, average monthly fishing is around 45 metric tons per month. The participation of Manchar Lake in the fishing industry is acknowledged as a main medium of livelihood for fish communities, and the activity seems to be more accessible [22]. The management of the marine industry was critical in increasing fishermen's families' food, nutrition security and livestock nourishment [23]. Fish and certain other seafood include a variety of nutrients, particularly fat, which have been shown to be essential for healthy development [24]. The importance of fishery resources for revenue production and alleviating poverty has been addressed in the latest studies [25].

### 2.2 Livelihood of Manchar Lake's fishermen

Ecological and social variations in reservoirs have ramifications for fishermen's livelihoods, forcing them to migrate and alter their vocation. When questioned about whether lakeside rehabilitation would also recover lost livelihoods, proponents of lakeside preservation sometimes find themselves in a quandary [26]. Because most individuals are not designed for shifts in their livelihood arrangements, attempting to respond to such issues is challenging. Fishing families' employment has undergone a full shift, with significant inequality in the availability of resources and livelihood prospects [27]. Despite improved access to physical capital such as schools, hospitals, transportation, and marketplaces, the communities remain exposed to financial instability and seldom enjoy benefits such as affordable housing. The status of one's present and previous livelihoods and asset holdings determines one's inclination to return to fishing in Manchar Lake. Given current economic improvement, most fishermen in the Manchar Lake region remain impoverished, surviving on less than two dollars per day (gross). The majority of coastal fishing households rely on marine sources of food and income [28], because most small-scale fishermen are uneducated and they are unable to work in other fields. In Dadu municipality, where Manchar Lake is located, the majority of fishing communities have no schooling, while 35.56% have finished elementary education. Fishermen live in substandard houses with substandard floors, walls, and roof building supplies. Metal sheeting is a prevalent construction material in around half of the buildings built with an earthen floor. Seventeen fishermen's residents live on their boats, which lack basic electrical and gas amenities, therefore, kerosene lamps are used for illumination and charcoal is mostly used for cooking [29]. Only 19 % of the fishermen possessed home furnishings such as a TV, refrigerator, stove, or iron, and therefore only 5 % had a motorcycle, according to land acquisition data. Fishermen at Manchar Lake, on the other hand, work well together in their villages and support one another, particularly during times of recession brought on by the fishing season's lack of abundance. They actively take part in community concerns such as the construction of schools and mosques, as well as other social and development initiatives. Pakistan's government has established a number of programs to help fishermen to improve their living conditions. With World Bank funding, the Pakistan Poverty Alleviation Fund (PPAF) began its Livelihood Enhancement and Protection (LEP) initiative in May 2009 with a main focus to improve economic productivity and opportunity for poor and ultra-poor households. Marine protected areas were established to protect critical habitats in mangrove ecosystems in lagoons, conserve sea grass and coral reefs, and enhance sustainable artisanal fisheries and agriculture practices. A new livelihoods model called Livelihoods, Employment, and Enterprise Development (LEED), which focuses across the poverty spectrum, was introduced in July 2013 [30].

### 2.3 Financial usability and livelihood assets

Accessibility of finance fluctuates based on an individual's actions or society's changing circumstances [31]. Although it is apparent that distress is not the sole reason for the shortage of funds, the socio-emotional aspects of the poverty study are critical [32]. Fishermen on the Manchar Lake are often impoverished, with little rights and access to livelihood assets such as land and other physical assets. The majority of Manchar's fishermen are the offspring of small-scale fishermen. The relevance of livelihood assets, such as physical, financial, human, social, and natural assets, has been underlined extensively in various studies. These livelihood assets are responsible for an expressive effect on the fishermen's proceeds and welfare [33]. Landing places, fishing equipment, and warehouse infrastructure such as refrigeration units, vessels, motors, and fishing lines are all essential for catching fish. Fishing vessels, nets, tools, and certain other commercial fishing facilities are the most important physical assets for fishermen [34]. Highways, reservoirs, residences, education institutions, marketplaces, hospitals, energy, and sewerage systems are examples of non-fisheries physical assets [35]. Multiple studies have found that the use of physical assets for fish farming has a significant impact on fishermen's income. Improved fish-friendly business environments, basic assistance, and warehousing facilities can improve the lives of local artisanal fishermen [36]. Physical capital appears to be linked to small-scale fishermen's livelihood results, according to existing research. Fishermen's access to physical assets almost always leads to an increase in family income [37]. Small-scale fishermen's livelihoods can be improved by utilizing borrowings and even other means of cash such as subsidies, cash reserves, and inheritance [38]. Poor fishermen, according to the literary works, do not have access to formal financial services [39], and because of their low income and collateral requirements, fishermen have negligible access to appropriate financing. Lending institutions are the primary source of capital for fishermen's families. Poor fishermen, on the other hand, are manipulated by shady moneylenders who charge exorbitant interest rates on their debts. Due to the relative discounted value of payback, many lenders are unwilling to lend funds to small-scale fishermen [40]. Human assets have quite a significant role in fishermen's financial wellbeing. Education levels, fishing knowledge and experience, and a physical capacity to work were used to assess this asset. Poor livelihood outcomes are linked to fishermen's limited knowledge, capabilities, and potential. A high-level skillset along with additional experience, as well as excellent health conditions, are required for the generation of high-income levels. Fishermen's abilities, knowledge, and skills might boost their chances of getting better employment. Fishermen, on the other hand, are less likely to have access to healthcare facilities due to their financial insufficiency. Fishing expertise aids fishermen in mitigating the potential negative impacts on natural resources. Fishing is the sole source of income for Manchar's fishermen. Other professions need the abilities that fishermen lack. As a result, they are unable to transition to other jobs to support themselves. Consequently, social assets are projected to provide advantages. Fishermen in the community build social capital from a combination of communication networks, trustworthiness, collaboration, and a sense of belonging. In addition, their income can be aided by social capital. According to prior findings, the sustainability of fisheries is influenced by the extent of the network and cooperation between the fishing industry and other institutions.

### 2.4 Fisheries management

The difficulty of integrating fishing and optimizing asset exploitation is attributed to the lack of execution of fisheries legislation and requirements. The key cause of small-scale fishermen decreasing is due to ecological outcomes with inadequate enforcement of a fisheries policy [41]. Biodiversity ought to be managed in order to maintain coastal ecosystems, guide sustainable resource use, and promote rule and regulation adherence [41]. People's conformity with regulatory requirements is essential to optimal management. To keep small-scale fisheries alive, comprehensive management of coastlines with configurable options is required [42]. All of those are crucial not only in terms of finance, such as a reliable significant source of seafood and jobs, but also in terms of the social fabric of coastal towns, such as excellent relationships between fishermen and fishing administrations. Marine resource management is an economically and culturally direct conversion defined by human lifestyles and beliefs, with policies and decision-making influenced by notions including both design and layout. There are several techniques to manage marine resources that are currently in use. The capacity to utilize and manage ocean resources in a sustainable manner is enhanced by centralized approaches like marine protected zones and enforcing fish harvest restrictions, which determine the health, efficiency, and adaptability of ecological systems. According to a number of studies, sociological, financial, and regulatory factors are the most important predictors of local involvement and also have a considerable influence on fishermen's productivity. Difficulties about the present condition of deterioration are integrated into a community-based strategy that ensures environmental services are maintained in a sustainable direction through community-driven activities with regard to food security, local employment, and income for local fishermen.

## 3 Theoretical basis and research hypothesis

### 3.1 Conceptual framework

To better evaluate the degree of vulnerability of fishermen's livelihood asset entitlements across livelihood income in Pakistan, we classify the factors associated with the following groups: 1) Human Capital Category: Physical capacity of fishermen is linked to abilities to create new assets and opportunities, education, knowledge, experience and observation, technical training, good health condition, fishermen's ability to work, and their families. 2) Social Capital Category: According to social capital determination, the way in which individual's household and community work together is very important for household livelihood. Effective collaboration is connected with credibility, connection, mutual support, relationships, friendship, and participation of a fisherman as a responsible participant, all of which can play a critical role, particularly in times of crisis. 3) Physical Capital Category: Fishing equipment, warehousing facilities, infrastructures (e.g., buildings, roads, and bridges), production equipment such as fishing vessels, and boating motors, are included in the physical asset's realization classification, although non-fishing properties have included accommodation, housing items, and possession of any motor vehicles. 4) Financial Capital Category: A source of the amount of funds accessible to fishermen through various sources, such as cash, investments, savings, credit facilities, pensions, and other subsidies. 5) Natural Capital Category: The way in which people have opportunity to exploit natural resources such as agricultural land, soil, water, air, forest and livestock resources, and fishing fleets; ecosystem benefits (hydrological cycle), and ecological diversification need to be considered as well as the condition of resources themselves, their productivity and how they may be changing over time. 6) Livelihood Income: This parameter is determined by the amount of money earned by fishermen through their fishing activity. Other non-fishing sources of revenue include local business, handiwork, and masonry. The sale of secondhand assets is the last source of revenue for Manchar Lake's fishermen.

**Fig. 1:**
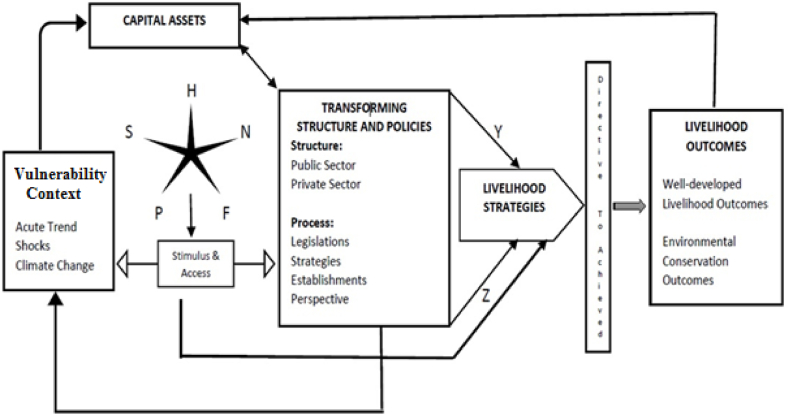
Sustainable livelihood framework.

The objectives of this study were: i) a principal component analysis approach to determine fishermen's revelations about their livelihood income.

ii) Assessment of change in livelihood income of fishermen's household's accordance to access of livelihood capital in the region. Based on our literature review, we developed the following hypothesis (Fig. 1).

**Fig. 2:**
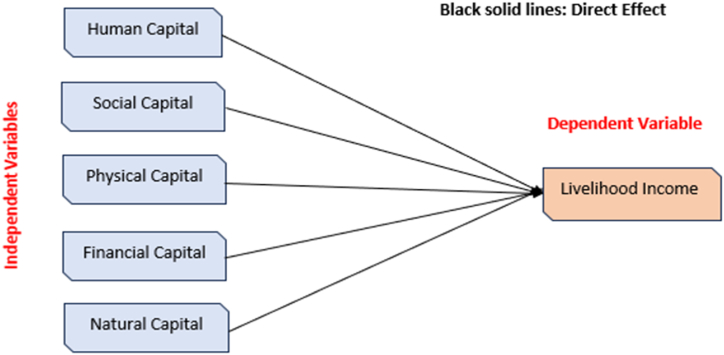
Conceptual framework of Study.

**Fig. 3:**
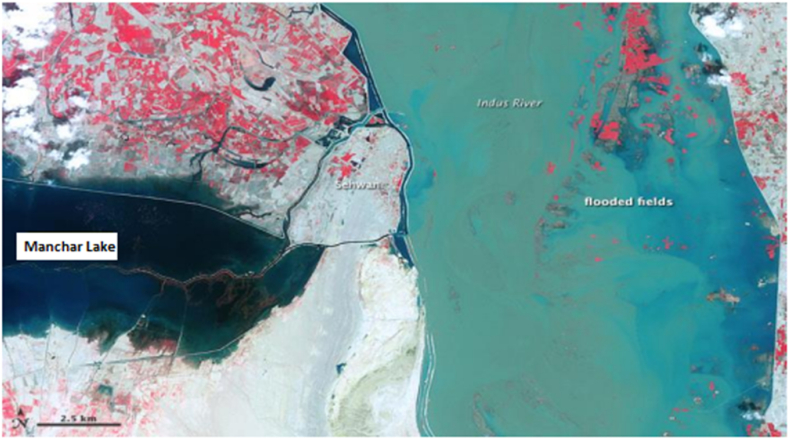
Map of manchar lake.

### 3.2 Research hypothesis

• **H1:** Human capital significantly influences household's livelihood income of fishermen.
• **H2:** Social capital significantly influences household's livelihood income of fishermen.
• **H3:** Physical capital significantly influences household's livelihood income of fishermen.
• **H4:** Financial capital significantly influences household's livelihood income of fishermen.
• **H5:** Natural capital significantly influences household's livelihood income of fishermen.

## 4 Materials and methodology

### 4.1 Study area and source of information

Sindh Province in Pakistan was used as the source of the data. In terms of fishing resources, Pakistan's Sindh Province is one of its richest. Nonetheless, this province remains an undeveloped region of Pakistan for sustainable livelihood options among fishermen due to the lack of infrastructural development and knowledge among the local fishermen. In order to examine the research model of this study, a survey approach was employed for data gathering. The locals living in the coastal region of Sindh Province's Manchar Lake are the study's target demographic. Three local indigenous people supported the author in the data collection procedure. The author initially met with the local community with the assistance of a native resident to explore the livelihood methods they employ and what effects these have on their socioeconomic elements of life in terms of the availability of livelihood assets. The full questionnaire was initially created in English before being translated into Urdu. English and Urdu are included in the questionnaire to help local residents comprehend the questions being asked. Fifty samples were used in the author's initial pilot survey, and once it was determined that the results were sufficient, these fifty samples were dropped from the final data collection. Using a random sample technique, we distributed 600 surveys in printed form to several Manchar Lake locations along with small gifts. There were 532 completed questionnaires received (after incomplete or unusable surveys were removed) which resulted in an 88.66 % response rate. Studies in the past have advised using a random sample strategy when the sample has distinct features and there is less information available [43]. According to demographic data for all samples, 67 % of participants were men and 33 % were women.

The respondents in this study were interested in the employment of different age groups in the fishing industry. All procedures performed in this study involving human participants were in accordance with the ethical standards of the institutional review board - China Three Gorges University (IRB2021SP001). All participants were made aware that their responses would be kept anonymous and confidential and that the data would only be utilized for the purposes of the study. Data used in research came from a survey that was conducted at landing areas from June 2021 to March 2022.

The survey measurements of the current study have been validated. A questionnaire was designed that consisted of different statements according to our study factors. Each statement reflects fishermen's perceptions about the subject matter. There were five possible options for each question, but respondents had to mark only one from the available five options. All the respondents were carefully instructed before filling out the questionnaire. The data was collected using a random data collection technique. Primary data collected from fishermen was used in this study. A sample was selected from the population of fishermen working and living near the Indus River, especially at Manchar Lake. In order to collect the data, a questionnaire was used that was developed for the realization of livelihood capital assets to ensure the upgrade of the fishermen's household income for the development of a sustainable livelihood for the Manchar Lake's fishermen in Pakistan. Validation of the research instrument involved three experts from relevant fields. There were three male experts, and their range of experience is between five and fifteen years. To measure the validation and reliability of the research instrument, a pilot study was conducted with fifty fishermen. The research instrument was improved on the basis of a pilot study, and verbal instructions were added to the data collection.

### 4.2 Assessment of livelihood capital assets

Following Pearson's advice from 1901, the assessment of livelihood assets used Principal Component Analysis (PCA) to combine several components into a single dimension [44] (see Table 1). When compared to simple summation, PCA is more accurate and relatively straightforward to perform. According to the ultimate theory of these methodologies, ownership of various assets causes a latent variable from each type of livelihood asset to show itself [45]. Principal component analysis was utilized in the study for two reasons: first, it is conceptually identical rotating the dimensions to reduce variation from the observation. Second, the amount of knowledge a variable imparts about other variables is reflected in its coefficient. An asset's ownership provides more or less information about certain assets when the correlation is positive or negative. Prior to employing a high number of linked components in another study, PCA is sometimes used to reduce them to a sensible size. The overarching goal of PCA is to keep enough of the data set's heterogeneity while reducing the information feature's multiplicity, which is composed of several connected components. When using principal component analysis, the component with the most Eigen values is chosen. You may create categorization while applying the algorithm: WI = RW/R, where WI represents the sum of the weighted index, R represents the percentage of influence of each selected variable, and R is the value given by the respondents for each variable. Each of the four aspects that made up the five categories of livelihood assets were evaluated using five parameters. The physical, financial, human, social, and natural assets that support a livelihood are included in this analysis. These assets are quantified by a set of variables that are outlined in Table 2. Throughout this analysis, the dependent variable was household income. For estimating household income, the paper conducted three components with five parameters for each category.

**Table 1:** Respondents’ profile.

Respondent Description	Frequency	Percentage
**Male**	356	67 %
**Female**	176	33 %
	**Age of respondent**	
**25**–**30**	146	27.35 %
**31–35**	221	41.56 %
**36–40**	68	12.84 %
**41–45**	97	18.25 %
	**Educational Details**	
**Did not complete primary school**	409	76.88 %
**Completed primary school**	123	23.12 %

**Table 2:** Variables’ categorization of different livelihood capital assets.

Dependent Variables	
Livelihood Income	*This parameter is determined by the amount of money earned by fishermen through their fishing activity. Other non-fishing sources of revenue include local business, handiwork, and masonry. The sale of the secondhand assets is the last source of revenue for Mancher Lake's fishermen.*
**Independent Variable**	
Human Capital Category	*Physical capacity of fishermen is linked to abilities to create new assets and opportunities, education, knowledge, experience and observation, technical training, a good health condition, and ability of fishermen to work and support their families.*
Social Capital Category	*According to social capital determination, the way in which individual's household and community work together is very important for household livelihood. Effective collaboration is connected with credibility, connection, mutual support, relationships, friendship, and the responsible participation of a fisherman, all of which can play a critical role, particularly in times of crisis.*
Physical Capital Category	*Fishing equipment, warehousing facilities, infrastructure (buildings, roads, bridges), production equipment such as fishing vessels, and boating motors are included in the physical asset's realization classification, although non-fishing properties have included an accommodation, housing items, and possession of any motor vehicle.*
Financial Capital Category	*A source of the amount of funds accessible to fishermen through various sources, such as cash, investments, savings, credit facilities, pensions, and other subsidies.*
Natural Capital Category	*The way in which people have opportunity to exploit natural resources such as agricultural land, soil, water, air, forest and livestock resources, fishing fleets, ecosystem benefits (hydrological cycle), and ecological diversification, need to be considered as well the condition of resources themselves, their productivity and how they may be changing over time.*

### 4.3 Regression model

To estimate the affiliations concerning small-scale fishermen's livelihood income and livelihood capital assets at Manchar Lake in Pakistan, the multiple regression analysis technique was utilized. The resultant variable, as illustrated in the model, is household income, which is affected by potential determinants such as descriptions of specific household assets such as human, social, physical, financial, and natural capital.

Yi = α + β1C1 + β2C2 + β3C3 + β4C4 + β4C5 + μe

Yi = fishermen's livelihood Income, C1 = human capital, C2 = social capital, C3 = physical capital, C4 = financial capital, C5 = natural capital and μe = unobserved variables. a, β1, β2, β3, β4, and β5 are constant factors to be considered.

## 5 Results and analysis

### 5.1 Findings of factor analysis

To create test indicators, principal component analysis is being used. Through Principal Component Analysis (PCA), components were retrieved again from the correlation matrix (PCA). The coefficient of determination shows how much variation a component may explain for a particular parameter. Each identified factor exclusively contains variables with factor loadings of p 0.5 and higher. The possibility that the statistics had enough relationship showing component assessment in determining which components had to be deleted; Bartlett's test of homogeneity of variance was also performed. To determine if the information taken is appropriate for the purpose of assessment, the Kaiser-Meyer-Olkin Score of Sampling Adequacy (KMO) is implemented.

#### 5.1.1 Household income category statistics

An outcome variable called household income includes three components: earnings from fishing, income from selling off assets, and income from non-fishing activities, which are shown in Table 3. The findings for these dimensions' factor loading values were higher than the suggested minimum value of 0.5. The KMO values for the components seem to have been 0.762, 0.743, and 0.692, correspondingly, showing that the statistics are appropriate for principal component analysis at this time. Consistent with those reported for Bartlett's measure of sampling adequacy test indicates that there is adequate correlation seen between components to move forward with regression analysis.

**Table 3:** Fisher's household income category.

No	Statements	Factor loading
1	Fishermen's families earn income through sources of fishing undertakings.	0.762
2	Fishermen's families earn income through sources of non-fishing undertakings.	0.743
3	Fishermen's families earn income through selling available property.	0.692

#### 5.1.2 Human Capital Category statistics

In Table 4, the four dimensions make up the variable known as human capital, including technical knowledge, fishing experience, fishermen's health condition, and innovative methods and tools of fishing displayed. The factor loading value findings revealed that all four dimensions were greater than the suggested threshold value of 0.5. The KMO values came in at 0.819, 0.630, 0.792, and 0.741, respectively. This conclusion suggests that Principal Component Analysis (PCA) can be performed on the available data. The results of Bartlett's measure of sampling adequacy were significant (p < 0.05), suggesting that there was enough correlation between the dimensions to move forward with the research.

**Table 4:** Human capital category.

No	Statements	Factor loading
1	Technical knowledge/education benefited the fishermen in catching great amounts of fish.	0.819
2	Fishing experiences have a significant influence on catching fishing.	0.630
3	Excellent health is necessary for fishermen for better fishing activities	0.792
4	Innovative methods and tools in fishing activities enhanced fishermen household's living standard.	0.741

#### 5.1.3 Social Capital Category statistics

The four dimensions make up the variable known as human capital, including technical knowledge, fishing experience, the fishermen's health condition, and innovative methods and tools of fishing displayed. The factor loading value findings revealed that all four dimensions were greater than the suggested threshold value of 0.5. The KMO values came in at 0.819, 0.630, 0.792, and 0.741, respectively. This conclusion suggests that Principal Component Analysis (PCA) can be performed on the available data. The results of Bartlett's measure of sampling adequacy were significant (p 0.05), suggesting that there was enough correlation between the dimensions to move forward with the research. Social capital is a variable that has four components shown in Table 5: social networking, credit worthiness, management, and citizen influence. Factor loading values for all dimensions were higher than the suggested threshold value of 0.5. The KMO produced findings of 0.707, 0.569, 0.588, and 0.732, respectively. This verdict suggests that Principal Component Analysis (PCA) was performed on the available data. The results of Bartlett's measure of sampling adequacy were significant (p < 0.05), suggesting that there was enough correlation between these dimensions to continue with the investigation.

**Table 5:** Social capital category.

No	Statements	Factor loading
1	Fishing operations could use a massive network of partners.	0.707
2	Fishermen's credit worthiness is a key element in fostering positive relationships in the fisheries sector.	0.596
3	The majority of fishermen strongly believe and trust in the fisheries management	0.588
4	Citizenship status allows more active contribution in fishermen's livelihood.	0.732

#### 5.1.4 Physical Capital Category statistics

Table 6 shows that storage facilities, fishing gear, housing and infrastructure factor loading values for these four dimensions were higher than the suggested minimum level value of 0.5. Therefore, nothing should be ignored. The KMO values of particular dimensions were 0.641, 0.702, 0.627, and 0.714, respectively, which indicates that Principal Component Analysis (PCA) was performed on the available data. Bartlett's measure of sampling adequacy test also revealed significant results (p < 0.05), demonstrating that there is enough of a significant relationship between these dimensions to move forward with the regression analysis.

**Table 6:** Physical capital category.

No	Statements	Factor loading
1	Fishermen are able to use storage facilities.	0.641
2	Fishing tools and gear are owned by fishermen that facilitate fishing activities.	0.702
3	The fishermen have their own housing amenities with some title of property.	0.627
4	Access of market services and Infrastructure influence a suitable fishermen's livelihood.	0.714

#### 5.1.5 Financial Capital Category statistics

As shown in Table 7, credit, savings, funds, and investment factors are the four dimensions that make up financial capital. As stated in Table 4, the results from the factor loading value of a certain dimension exceeded the advised threshold value of 0.5. This outcome points to the importance of factors that cannot be overlooked in the analysis. Specific dimensions' KMO values were 0.546, 0.592, 0.608, and 0.693, respectively. This KMO result shows that the current data is used for principal component analysis. Similar results were reported for Bartlett's measure of sampling adequacy, demonstrating a strong enough correlation between the two dimensions to move forward with the study (p < 0.05).

**Table 7:** Financial capital category.

No	Statements	Factor loading
1	Availability of credit facilities and subsidies enriched fishermen activities.	0.546
2	Fishermen's savings account ownership provides for the fishermen's family.	0.592
3	The possession of funds for everyday spending accessible for household usage improves fishermen's livelihood.	0.608
4	Investment from savings in other activities aids in fishermen's livelihoods in periods of low fishing production.	0.693

#### 5.1.6 Natural Capital Category statistics

Shown in Table 8 are land acquisition, ecosystems, livestock husbandry, biodiversity, and they make up the four dimensions of natural capital. The factor loading findings for all categories were higher than the suggested threshold value of 0.5. Here, 0.641, 0.755, 0.690, and 0.788 were the respective KMO values. This finding suggests that Principal Component Analysis (PCA) may be performed on the available data. The results of Bartlett's measure of sampling adequacy were significant (p < 0.05), suggesting that there was enough correlation between these dimensions to carry on with the investigation.

**Table 8:** Natural capital category.

No	Statements	Factor loading
1	Land acquisition is an essential significant element of fishermen's livelihood	0.641
2	For substantial fish stocks, the ecosystem is critical for fisherman's households.	0.755
3	Commercial fishermen benefit financially from livestock husbandry during socioeconomic downturns.	0.690
4	Biodiversity lifts ecology productivity of fisheries species.	0.788

### 5.2 Findings of regression analysis

The research findings suggest that positive and statistically significant coefficients for financial capital, human capital, social capital, physical capital, and natural capital are associated with income levels for fishermen. The considerable direct association between income and assets supporting a livelihood suggests that inland communities around Manchar Lake's accessibility to such assets increased their earnings. The regression findings show that small-scale fishermen's access to financial resources has greatly improved (credit availability from different sources, subsidies, savings, and investments). Their annual income was influenced by their availability of financial resources. According to the findings, improved access to human capital (training, knowledge, skills, and health) has greatly increased for fishermen's families. The availability of human capital greatly increased the family income of fishermen. The findings show that families' use of natural capital has greatly strengthened (fishing grounds, ecosystem services, livestock, and biodiversity). The economic status of fishermen has certainly benefited from access to natural capital. The findings also show that fishermen now have considerably better access to social capital (social networking, credit worthiness, relationships, trust, citizen influence, management, and partnership). Regarding fishermen in the Manchar Lake Basin, access to social capital has a sizable influence on household income (Table 9). Although higher, the physical capital coefficient is vanishingly small. The findings showed that the poor fishermen's degree of physical capital (fishing equipment, boats, gear, storage facilities, and market amenities) has become too low to have a meaningful influence on the growth of their income (see Table 10).

**Table 9:** Correlation between Household Income of fishermen and Livelihood Capital Assets.

Variables	B	Std. Error	t-value	p-value
(Constant)	2.138	0.769	2.780	0.006
Human capital	0.267	0.034	7.960	0.000
Social capital	0.180	0.027	6.610	0.000
Physical capital	−0.079	0.032	−2.425	0.016
Financial capital	0.097	0.026	3.807	0.000
Natural capital	0.021	0.030	0.690	0.049
R-Square	0.545			
Adj- R Square	0.536			
F- Ratio	41.790			
F- Probability	0.000

**Table 10:** Results of hypothesis tests.

Hypothesis	Description	B	R^2^	F	T-value	P-value	Hypothesis
**H**_**1**_	Significant influence of human capital on household's livelihood income	0.267	0.545	41.790	7.960	0.000	Accepted
**H**_**2**_	Significant influence of social capital on household's livelihood income	0.180	0.545	41.790	6.610	0.000	Accepted
**H**_**3**_	Significant influence of physical capital on household's livelihood income	−0.079	0.545	41.790	2.425	0.016	Accepted
**H**_**4**_	Significant influence of financial capital on household's livelihood income	0.097	0.545	41.790	3.807	0.000	Accepted
**H**_**5**_	Significant influence of natural capital on household's livelihood income	0.021	0.545	41.790	0.690	0.049	Accepted

## 6 Discussion

The results of the study indicated that human capital access has significantly contributed to the dependent variable household income of fishermen, which was regressed on the predicting variable human capital to test the hypothesis H1- > HC: (Human capital significantly influences the household livelihood income of fishermen). Human capital significantly predicted F = 41.790, p < 0.001, which indicates that human capital can play a significant role in shaping household income (b = 0.267, p < 0.001). Therefore, the result clearly directs the positive relationship between proposed hypothesis of fishermen's household income and access of human capital was accepted. Moreover, the R^2^ = 0.545 indicates that the model explains 54.5 % of variance in household income. Table 9 shows the summary of the findings. The findings are in line with those of earlier research, which concluded that an absence of adequate academic achievement does not always translate into a lack of fishing expertise or experience. The bulk of fishermen have long histories of fishing activity, which has given them substantial environmental expertise. The findings, however, are not confirmed by other research, which revealed that small-scale fishermen earned so little from fishing and were unable to switch to adequate occupations because they lacked education.

The hypothesis tests if social capital carries a significant impact on fishermen's household income. The dependent variable household income was regressed on the predicting variable Social Capital to test the hypothesis H_2_–>SC (social capital has a significant influence on household livelihood incomes of fishermen). Social capital significantly predicted Household Income, F = 41.790, p < 0.001, which indicates that social capital can play a significant role in shaping Household Income (b = 0.180, p < 0.001). Therefore, the result clearly directs the positive relationship between the proposed hypothesis of fishermen's income and access of social capital was accepted. Moreover, the R^2^ = 0.545 indicates that the model explains 54.5 % of variance in household income. According to the study's findings, there is a strong and positive correlation between fishermen's household income and social capital. As a result, the hypothesis that was presented was validated by showing that there is a positive correlation between fisherman's income and their ability to access social capital. The findings of those who noticed that coastal regions prefer to lend a hand to one another amid economic difficulties reflect these findings. The findings differ from those of another analysis, which revealed that disputes between fishermen regarding fishing sites and fish markets occur often.

The hypothesis tests if physical capital carries a significant impact on fishermen's household income. The dependent variable household income was regressed on predicting variable physical capital to test the hypothesis H_3_–>PC: (physical capital significantly influences household's livelihood income of fishermen). Physical capital significantly predicted household income, F = 41.790, p < 0.001, which indicates that physical capital can play a significant role in shaping household income (b = 0.097, p < 0.001). Therefore, the result clearly shows that the positive relationship between proposed hypothesis of fishermen's income and access of capital was accepted. Moreover, the R^2^ = 0.545 depicts that the model explains 54.5 % of variance in household income. The findings shows that it somewhat mediates for natural capital, social capital, human capital, and physical capital. However, norms and regulations relating to financial capital and fishermen's revenue fully mediated the financial capital.

The hypothesis tests if financial capital carries a significant impact on fishermen's household income. The dependent variable household income was regressed on predicting variable financial capital to test the hypothesis H_4_–>FC: (Financial capital can significantly influence the household's livelihood income of fishermen). Financial capital significantly predicted household income, F = 41.790, p < 0.001, which indicates that the financial capital can play a significant role in shaping household income (b = −0.079, p < 0.061). Therefore, the result clearly shows the positive relationship between proposed hypothesis of fishermen's income and access of financial capital was accepted. Moreover, the R^2^ = 0.545 depicts that the model explains 54.5 % of variance in household income. The findings of the financial capital study imply that the household income of fishermen has a positive and substantial link with access to various financial assets. As a result, the presented hypothesis showed that there is a positive correlation between fishermen's income and their ability to access financial capital and was accepted. This outcome is consistent with the previous study's results that fishermen's livelihoods were enhanced by having access to funds. The fishing industry's credit system supports and promotes fishing. The study's findings, which do not align with those of previous research, indicate that credit did not help impoverished fishermen escape poverty. Access of finance only results in short-term utilization and stabilization with a risk of becoming caught in an endless cycle of debt, not long-term optimization of lifetime opportunities.

The hypothesis tests if natural capital carries a significant impact on fishermen's household income. The dependent variable household income was regressed on predicting variable natural capital to test the hypothesis H_5_ –>NC: (natural capital can significantly influence the household's livelihood income of fishermen). Natural capital significantly predicted household income, F = 41.790, p < 0.001, which indicates that the NC can play a significant role in shaping household income (b = 0.021, p < 0.049). Therefore, the result clearly shows that the positive relationship between proposed hypothesis of fishermen's income and access of natural capital was accepted. Moreover, the R^2^ = 0.545 depicts that the model explains 54.5 % of variance in household income. The study found a strong and positive correlation between household income and natural capital among fishermen. The suggested association between the income of fishermen and their access to natural capital was recognized. The findings are consistent with those of another study. Despite the abundance of fish stocks in the fishing grounds, modern fishing equipment is still necessary for considerable fish catches. This conclusion, meanwhile, was at odds with that of researchers who revealed that human activity is causing fishing grounds to deteriorate, which is why fish catches are dropping.

Additionally, this study examined how fishing laws and restrictions affected the family income of fishermen in Manchar Lake as a mediator. In order to improve lives and the economy, the blue economy has been designated as a key issue for the next 20 years in the Manchar Lake Development Vision. The report makes policy recommendations to the government for developing a blue economy while taking into account the contribution of the fishing industry. By designating fishing zones between them and medium-scale fishers, Manchar Lake's small-scale fishermen should be safeguarded against migrant fishermen. The fish market has to be upgraded with facilities, including clean water, storage capacity, and services for fish processing. Providing them with technical and financial support, the government should give the fishermen more influence. Fisheries may be a priority for local investors since it is an opportunity to invest that is still continuing even though the rewards seem promising. In order to maximize the prices in terms of revenue for the fishermen and the economic growth of Manchar Lake, the food manufacturing sector is required.

## 7 Conclusion and recommendations

### 7.1 Conclusion

The research examines the socioeconomic issues facing the fishing community in Manchar Lake's coastline region. Data on a variety of dimensions, including household income sources and assets ownership, were gathered from a sample of fishing families. Structured questionnaires were used to gather primary data, while public government reports were used to gather secondary data on socioeconomic status. The significant livelihood resources that provide household advantages are recognized and examined. Multiple regression analysis explains the relationships between household assets and household income factors. According to the survey, Manchar Lake's fishing settlements are among the poorest in the country. Their primary sources of income are fishing and the sale of labor at cheap rates. The study's findings imply that fishermen's access to fishing equipment, including gear and vessels, is crucial. Instead of the current situation of splitting the profits between the fishermen and the owner of the fishing equipment, if fishermen have complete ownership of their fishing properties, they may guarantee the entire income from fish sales. For current fishing techniques and equipment, financial resources like the availability of credit facilities, subsidies, or grants are essential. Technical expertise, training in new fisheries technology, and the best methods for safeguarding coral reefs and mangroves are highly essential and can improve fishermen's fishing prowess and understanding. The livelihood of fishermen households depends greatly on social links, networking, citizen engagement in governance, and partnerships between the fishing community and other social groups. This research advises fishermen effectively engage in other economic activities, including farming, raising animals, running small enterprises, and investing in alternative occupations. The administration should prioritize the need to review the fisheries legislation and rules in order to compete with the current economic situation, as well as the need to devise a policy about the conservation of fisheries ecology and biodiversity for the future economic security of Manchar Lake's fishermen.

### 7.2 Recommendations

Understanding how vulnerable households in the poor community of fishermen on Manchar Lake currently access capital assets for their means of subsistence is crucial, therefore, the federal and local governments need to legislate rules and regulations to help this group. Distinct livelihood pursuits have different prerequisites. They have a variety of alternatives to select from in order to optimize the benefits of successful lifestyle choices, rather than being compelled to choose a certain technique because it is the only one accessible. By considering all relevant factors, the family income of small-scale fishermen in Manchar Lakeside must be increased. Consequently, the current regime should either take into account how coastal regions live in a variety of different circumstances, most of which are offered by offering socioeconomic and other financial support, such as promoting alternate livelihood security, coordinating collaboration, and integrating power structures, between them so that fishermen's livelihoods can improve. Based on the results of this analysis, it is clear that improving access to high-quality knowledge and competence development for the next generation is necessary to help this population make healthy choices. In order to boost production and revenue, self-employed families, including producers, fishermen, and smallholders, would be urged to use contemporary technologies' national level policy of sustainable development [11] of the fisheries sector and modification for vulnerability of livelihood income is much needed. Initiatives for developing long-term sustainability that use contemporary practices, as well as methodologies, need to be intensified. Local government should focus on promoting financial inclusion in lakeside settlements in order to grow financial assets of poverty-stricken fishermen. The plan places emphasis on fostering favorable domestic investment skills and the expansion of indigenous businesses. In order to encourage fishermen to give up fishing output and take on non-fishing endeavors and achieve a market existence, state institutions, government representatives and national level administrative agencies, should also aggressively embrace efficient measures of financial assistance. The government should encourage development of the fisheries sector at the domestic and national level in order to improve the income of local fishermen as well as national income. This will help shape the future of the livelihood income's potential benefits at the domestic and national level. Pakistan's government must develop policies for wellness of fishermen to improve their livelihood income.

## Data availability statement

Data will be made available on request.

## Funding statement

This work was supported by the 10.13039/100014717National Natural Science Foundation of China（72271142, Youth Fund for Humanities and Social Sciences Research of the Ministry of Education of China（19YJCZH264）and Excellent Young and Middle-aged Science and Technology Innovation Team Program for Higher Education Institutions of Hubei Province（T2022006）. This research did not receive any specific grant from funding agencies in the public, commercial, or not-for-profit sectors.

## Additional information

No additional information is available for this paper.

## CRediT authorship contribution statement

**Zhao Xu:** Supervision, Investigation. **Maria Qayum:** Writing – original draft, Software, Methodology. **Jamil Afzal:** Writing – review & editing, Writing – original draft. **Muhammad Aslam:** Writing – review & editing, Methodology.

## Declaration of competing interest

No conflict of interest regarding the paper.
